# Retraction: Examining the convergence of dominant themes related to social entrepreneurship, NGOs and globalization–A systematic literature review

**DOI:** 10.1371/journal.pone.0359051

**Published:** 2026-09-24

**Authors:** The PLOS One Editors

The *PLOS One* Editors retract this article [1] because it has come to light that the article’s peer review process was compromised.

MRH, KBM, SAM, and EM did not agree with the retraction. GNS and SO either did not respond directly or could not be reached.
